# Nailfold Capillaroscopic Changes in Children with Atopic Dermatitis

**DOI:** 10.3390/jcm15145395

**Published:** 2026-07-09

**Authors:** Zuhal Karali, Tuba Kurt

**Affiliations:** 1 Department of Pediatric Immunology and Allergy, Bursa City Hospital, Bursa Faculty of Medicine, University of Health Sciences, 16250 Bursa, Turkey; 2 Department of Pediatric Rheumatology, Bursa City Hospital, Bursa Faculty of Medicine, University of Health Sciences, 16250 Bursa, Turkey; drtubakurt@hotmail.com

**Keywords:** atopic dermatitis, nailfold capillaroscopy, children

## Abstract

**Background:** This study aimed to evaluate microvascular changes using nailfold capillaroscopy (NFC) in children with atopic dermatitis (AD) and to contribute to the understanding of the disease’s pathophysiology. **Methods:** The study included 71 children diagnosed with AD (33 girls/38 boys, median age 108 months) and 71 healthy controls (40 girls/31 boys, median age 126 months). Disease severity in AD patients was assessed using the SCORing of atopic dermatitis (SCORAD) index. Parameters with NFC evaluated included capillary density, size, morphology (tortuous, bushy), microhemorrhages, avascular areas, shortening, and crossed capillaries. **Results:** In the atopic dermatitis group, capillary shortening (<100 micrometers) was significantly more common in the AD group (33.8% vs. 8.4%, *p* < 0.001; OR = 5.53, 95% CI 2.10–14.59), representing the most robust discriminating finding. Tortuous capillaries (>10%) showed a higher prevalence in AD (36.6% vs. 22.5%, Fisher’s *p* = 0.04); however, this difference did not retain statistical significance after age adjustment (ANCOVA *p* = 0.076). Bushy capillaries were observed exclusively in the AD group (8.4% vs. 0%, Fisher’s *p* = 0.01), whereas microhemorrhages were detected only in controls (8.4% vs. 0%, Fisher’s *p* = 0.02). Given the wide confidence intervals, these findings should be considered exploratory. No significant differences were found in mean capillary count, dilated capillaries, giant capillaries, avascular areas, or crossed capillaries. NFC findings showed no significant correlation with the SCORAD index in mild and moderate AD. In the severe AD subgroup (*n* = 3), capillary shortening was observed in all three patients; however, given the very small sample size, this observation should be interpreted as preliminary and exploratory. **Conclusions:** Our study demonstrates that children with AD exhibit characteristic nailfold capillaroscopic changes, most notably reduced capillary length as the most robust discriminating finding, accompanied by the presence of bushy capillaries. Investigating the molecular mechanisms underlying these microvascular changes and their impact on disease prognosis will provide insight into future treatment strategies.

## 1. Introduction

Atopic dermatitis (AD) is a chronic inflammatory skin disease characterized by intense pruritus, eczematous lesions, and a relapsing course [1,2]. It is seen in approximately 20% of children, and due to this increase in incidence, AD is an important public health problem [3,4]. Genetic predisposition, immunological dysfunction, skin barrier dysfunction, and environmental factors are involved in the pathophysiology of AD. This dysregulation in the immune system, particularly the activation of type 2 lymphocytes, leads to deterioration of the epidermal barrier [5,6,7]. Epidermal barrier defects and immune regulation disorders, as well as vascular phenomena such as microvascular changes and angiogenesis, are involved in the pathogenesis [8,9]. Atopic dermatitis skin inflammation appears to be linked to vascular changes. Atopic dermatitis keratinocytes release pro-angiogenic factors such as vascular endothelial growth factor, while Th17 cells trigger angiogenesis via IL-17 [10].

Vascular dysregulation in atopic dermatitis has been associated with disease activity and symptom severity [11]. In the 3D morphology of peripheral blood vessels, thickened and tortuous vessels with increased blood flow were found in both lesional and non-lesional skin [12]. This situation reveals that atopic dermatitis is not just a skin disease but a complex disease accompanied by processes such as immune dysregulation and vascular remodeling [13].

Capillaroscopy is a crucial technique that assesses microvascular circulation by directly and non-invasively examining the nailfold capillaries [8,14]. Considering the chronic inflammation and angiogenic processes associated with atopic dermatitis, capillaroscopy may have diagnostic and prognostic value in detecting microvascular abnormalities in this disease, assessing disease severity, selecting treatment, and evaluating response to treatment [8].

Our aim in this study is to evaluate microvascular changes in children with atopic dermatitis using the nailfold capillaroscopy (NFC) method and to make significant contributions to the understanding of the disease’s pathophysiology.

## 2. Materials and Methods

**Figure 1:**
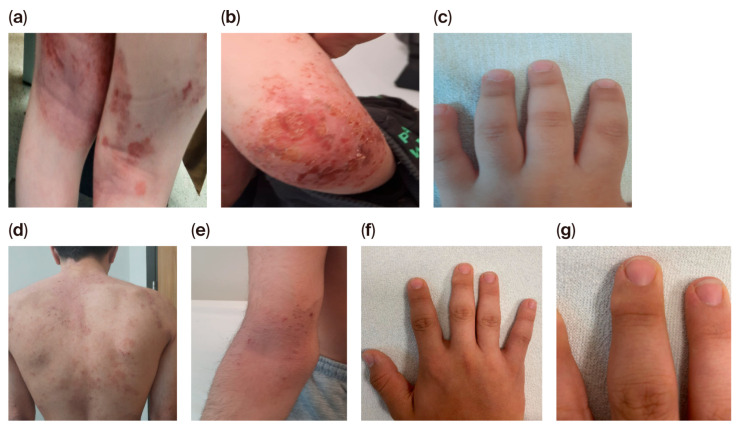
Representative clinical photographs of atopic dermatitis patients demonstrating the distinction between affected body regions and the clinically uninvolved nailfold examination site. Figures (**a**–**c**) represent images obtained from the same patient. Despite the presence of widespread atopic dermatitis lesions affecting other body regions, the dorsal hand view of the same patient demonstrated no evidence of active dermatitis, erythema, scaling, or excoriation in the nailfold and periungual examination areas. The periungual and nailfold skin remained intact and was therefore considered suitable for capillaroscopic evaluation. Figures (**d**–**g**) present dermatitis lesions from a different patient. Similarly, no lesions were observed in the hand and nailfold examination regions of this patient, confirming that the areas used for capillaroscopic evaluation were clinically unaffected.

Seventy-one patients who followed up at the pediatric allergy-immunology outpatient clinic between January 2024 and December 2024 and were diagnosed with atopic dermatitis were included in the study. Patients under 5 years of age; those with Raynaud’s phenomenon, a systemic disease (such as diabetes mellitus, systemic hypertension, thyroid diseases, etc.); those who used topical corticosteroids, topical and/or systemic antihistamines, or topical calcineurin inhibitors within the last month; and those who used systemic corticosteroids, immunosuppressants, and/or biological treatments within the last 6 months were excluded from the study. Patients with clinically active dermatitis lesions involving the hands, particularly the nailfold examination area, identified during physical examination by a pediatric allergist and assessed using the Scoring Atopic Dermatitis (SCORAD) index, were excluded from the study. Clinically active lesions were defined by the presence of pruritus, erythema, scaling, papules, edema, exudation/crusting, or excoriation. These patients were excluded because active inflammatory changes in the nailfold region could interfere with the acquisition of reliable capillaroscopic images and compromise accurate interpretation of the findings. In contrast, patients with active atopic dermatitis lesions located on body sites other than the hands and nailfold region were eligible for inclusion in the study. Atopic dermatitis was diagnosed according to Hanifin-Rajka criteria [15]. Representative clinical photographs demonstrating the macroscopic appearance of the examination site are presented in Figure 1. Despite the presence of widespread atopic dermatitis lesions affecting other body regions (Figure 1a,b,d,e), the dorsal hands and periungual skin of the patients were free of active dermatitis, erythema, scaling, excoriation, or nail abnormalities (Figure 1c,f,g), confirming the suitability of the nailfold region for reliable capillaroscopic evaluation. These findings visually support the clinical rationale underlying our exclusion criterion, whereby patients with active dermatitis involving the capillaroscopy examination area were excluded, while those with lesions confined to other body regions were eligible for inclusion.

Additionally, 71 healthy children with no known history of allergic diseases or clinical symptoms suggestive of atopy, and no diagnosed systemic disease, were included in the study. Disease severity in patients with atopic dermatitis was calculated by a pediatric allergist using the SCORAD index. Mild was defined as 0–25, moderate as 25–50, and severe as >50. Patients’ immunoglobulin levels, specific IgE levels, and skin prick tests were analyzed. Lymphocyte subsets were studied in patients at risk for immunodeficiency. Serum immunoglobulin levels were measured by immunoturbidimetric assay on a cobas 8000 modular analyzer (Roche Diagnostics, Mannheim, Germany). Specific IgE measurements were performed using the IMMULITE® immunoassay method (Siemens, Horseheads, NY, USA), and a cut-off value of 0.35 kU/L was used to define allergic sensitization. Skin prick testing was performed in the pediatric allergy clinic test room using stand-ardized allergen extracts (Diater Laboratorios, Madrid, Spain).

**Figure 2:**
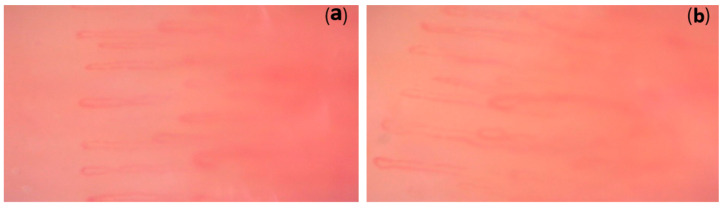
(**a**,**b**) Normal nailfold capillary appearance showing regularly arranged, parallel, and homogeneously distributed capillaries.

Because nailfold capillaroscopic evaluation cannot be performed reliably in children younger than five years of age, only patients aged five years and older with a diagnosis of atopic dermatitis were included in the study. Nailfold capillaroscopy was performed using a digital videocapillaroscope (CapillaryScope, Dino-Lite Medical, AnMo Electronics Corp., Taipei, Taiwan). Nailfold capillaroscopy evaluation of all patients and healthy children were performed by the same pediatric rheumatologist, and the resulting images were analyzed. The pediatric rheumatologist evaluating the capillary images was unaware of the patients’ clinical and laboratory data. A foreign body (nail polish, paint) removal and nail cutting were performed on all patients two weeks prior to the procedure. Prior to the procedure, the patients were kept at room temperature for 15 to 30 min. Patients with local trauma and thick nail fold skin were not evaluated. Sweet almond oil was used in conjunction with a 200-magnification capillaroscopy instrument to improve image quality. NFC was applied to four fingers, except the first finger of each hand. From each finger, at least four pictures were captured (Figure 2).

Since specific nailfold capillaroscopic evaluation criteria for patients with atopic dermatitis have not yet been established, this study applied the criteria proposed by the European Alliance of Associations for Rheumatology Microcirculation Study Group (EULAR), considering reference evaluations performed in healthy children [16,17].

The parameters assessed in NFC were capillary density (number of capillaries per linear mm), capillary size (diameter of the apical branch of the capillary), capillary morphology (shape of each capillary), and the presence or absence of microhemorrhages. Enlarged capillaries are represented by a diameter of >20 µm and <50 µm. Giant capillaries are defined as uniformly dilated, normally shaped capillaries with an apical diameter of 50 µm. Semi-quantitative rating scale (0: no change, 1: less than 33% capillary changes/reduction, 2: 33–66% capillary changes/reduction, 3: >66% capillary changes/reduction) was used. Capillaries shorter than 100 µm were classified as shortened capillaries, and those longer than 300 µm as elongated capillaries. An increase in convoluted capillaries and crossings was deemed noteworthy if it exceeded 10%.

### 2.1. Ethical Approval

Ethical approval was obtained from the University of Health Sciences, Bursa Faculty of Medicine, and the Bursa City Hospital ethics committee for the study (Approval No. 2024–9/12).

### 2.2. Statistical Analysis

Statistical analysis was performed using SPSS software version 22.0. The categorical variables were determined as number and percentage. The quantitative variables were evaluated using the Shapiro–Wilk test and a histogram to define whether they were normally distributed. Non-normally distributed variables were presented with median and interquartile range (IQR) values. The differences between categorical data were analyzed by using the χ^2^ test or Fisher’s exact test, between non-parametric independent groups by the Mann–Whitney U-test. The statistical significance level was pre-determined as *p* < 0.05. When comparing the mean differences between groups, covariate analysis (ANCOVA test) was used to determine the effect of additional variables (covariates) influencing the dependent variable. Fisher’s exact test was used as the primary inferential method for categorical intergroup comparisons. Odds ratios (ORs) with 95% confidence intervals (CIs) were calculated as supplementary effect size measures. For parameters with zero-cell counts, OR confidence intervals derived from the Haldane–Anscombe correction should be interpreted with caution, given the inherent instability of effect size estimation under sparse-data conditions. For contingency tables containing zero-cell counts, the Haldane–Anscombe correction (addition of 0.5 to all cells) was applied to allow OR estimation.

## 3. Results

Seventy-one (33F/38M) AD and (40F/31M) 71 healthy children were included in the study. Median age in children with AD and healthy children was 108 (89–139) and 126 (40–148) months, respectively, with no significant difference between groups (*p* = 0.9). The time to diagnosis was 24 months (range, 12–48 months). Fifty-three (74.6%) of patients with AD had allergic sensitization. Twenty-one patients (29.6%) had allergic rhinitis, 3 (4.2%) had asthma, 5 (7%) had urticaria, and 2 (2.8%) had both allergic rhinitis and asthma. In addition, 7 (9.9%) of these patients had a family history of AD. The median SCORAD index was 32.5 (23–39). Atopic dermatitis was mild in 21 (29.2%) patients, moderate in 47 (66.1%), and severe in 3 (4.2%) of the patients.

### 3.1. NFC Findings

**Figure 3:**
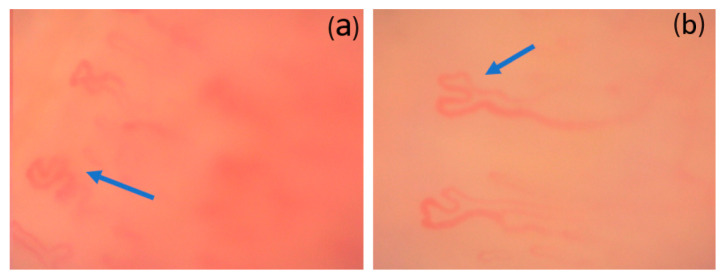
(**a**) Tortuous capillary pattern. (**b**) Bushy capillary appearance on nailfold capillaroscopy. Irregular capillary distribution is evident in both images. Blue arrows indicate the representative capillary abnormalities described in each panel.

**Figure 4:**
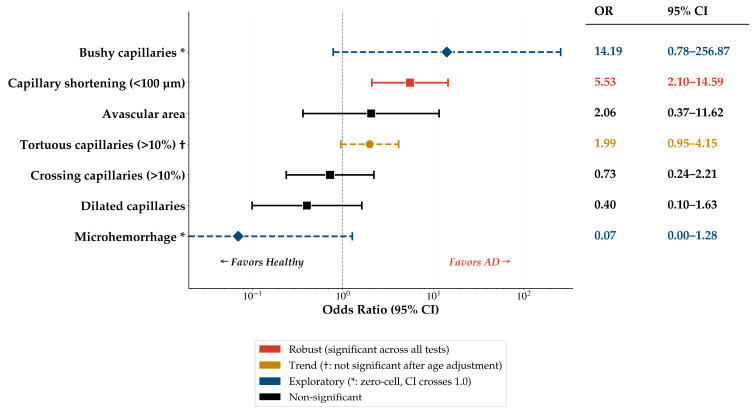
Forest plot of odds ratios (OR) with 95% confidence intervals (CI) for nailfold capillaroscopy findings in children with atopic dermatitis (*n* = 71) and healthy controls (*n* = 71). Findings were classified into four interpretive categories based on the consistency of statistical evidence across analytical methods (Fisher’s exact test, age-adjusted ANCOVA, and OR confidence intervals): (1) Robust finding (red): capillary shortening showed a strong association with AD across all analytical approaches (OR = 5.53, 95% CI 2.10–14.59), with the confidence interval clearly above 1.0, representing the most discriminating parameter. (2) Trend (yellow, marked with †): tortuous capillaries showed a higher prevalence in the AD group before age adjustment (OR = 1.99); however, the OR confidence interval (0.95–4.15) crosses 1.0, and the association did not retain statistical significance after age adjustment. This finding therefore represents a trend rather than a definitive association. (3) Exploratory findings (blue, marked with *): bushy capillaries and microhemorrhage showed group differences in favor of the AD and healthy control groups, respectively (OR = 14.19, 95% CI 0.78–256.87 and OR = 0.07, 95% CI 0.00–1.28). However, both parameters had zero observations in one of the groups and required the Haldane–Anscombe correction for effect size estimation. The resulting wide confidence intervals crossing 1.0 reflect substantial instability in the estimated effect sizes under sparse-data conditions. These findings should therefore be interpreted as exploratory observations rather than statistically confirmed associations. (4) Non-significant findings (black): avascular area, dilated capillaries, and crossing capillaries showed no group difference, with OR confidence intervals including 1.0. Squares, circles, and diamonds represent OR point estimates; horizontal lines represent 95% CIs; the dashed vertical line denotes OR = 1.0 (null effect).

The mean capillary count in patients with and without AD was 9.6 ± 1.7 and 10.6 ± 1.3, respectively, with no statistically significant difference in capillary count (*p* = 0.07). No correlation was found between the time until diagnosis and NFC findings (enlarged capillaries, microhemorrhage, capillary length, crossing and tortuosity increase, and avascular area) (*p* = 0.07, *p* = 0.7, *p* = 0.9, *p* = 0.7, *p* = 0.6, *p* = 0.3, *p* = 0.1). Enlarged capillaries were present in 3 (4.2%) patients in the AD group and in 7 (9.8%) patients in the healthy group (HG) (*p* = 0.3). The giant capillary was not present in both groups. An increase of more than 10% in the number of tortuous capillaries was detected in 26 (36.6%) patients in AD and 16 (22.5%) patients in HG. The microhemorrhage was present in 6 (8.4%) patients with HG and in no patients with AD. Bushy capillaries were present in 6 (8.4%) patients with AD and absent in HG (Figure 3). More than 33% of the avascular area was present in 4 (5.6%) patients in AD and 2 (2.8%) patients in HG. Shortening of capillary length (<100 micrometers) was detected in 24 (33.8%) patients with AD and in 6 (8.4%) patients with HG. The presence of short capillaries was significantly higher in AD (*p* < 0.001). The difference in length was evaluated between the AD and HG, and the mean length value of the HG (1.03) was found to be higher than that of the atopic dermatitis group (0.70). Nailfold capillaroscopy findings are shown in Table 1. To further quantify the strength of association between individual capillaroscopic findings and atopic dermatitis, odds ratios were calculated and are presented in Figure 4. Capillary shortening (<100 µm) demonstrated the strongest statistically significant association with AD (OR = 5.53, 95% CI 2.10–14.59, *p* < 0.001), indicating that children with AD were approximately 5.5 times more likely to exhibit shortened capillaries compared to healthy controls. Tortuous capillaries (>10%) were more prevalent in the AD group (OR = 1.99, 95% CI 0.95–4.15, Fisher’s exact test *p* = 0.04); however, this association did not retain statistical significance after age adjustment (ANCOVA *p* = 0.076), suggesting a trend rather than a definitive finding. Bushy capillaries were observed exclusively in the AD group (8.4% vs. 0%, Fisher’s exact test *p* = 0.01). The Haldane–Anscombe-corrected OR was 14.19 (95% CI 0.78–256.87). Microhemorrhage was observed exclusively in the healthy control group (0% vs. 8.4%; Fisher’s exact test, *p* = 0.02). The corrected OR was 0.07 (95% CI 0.00–1.28). Nevertheless, the wide confidence intervals that cross 1.0 for both findings reflect the instability of effect size estimation in the presence of zero-cell counts; therefore, these observations should be considered exploratory and interpreted with caution. No significant associations were found for dilated capillaries (OR = 0.40, 95% CI 0.10–1.63, *p* = 0.30), avascular areas (OR = 2.06, 95% CI 0.37–11.62, *p* = 0.40), or crossing capillaries (OR = 0.73, 95% CI 0.24–2.21, *p* = 0.70).

**Figure 5:**
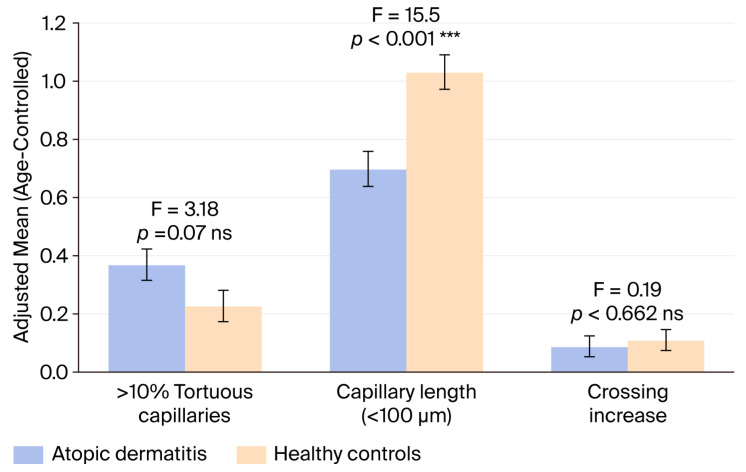
Age-adjusted mean values of nailfold capillaroscopy parameters in children with atopic dermatitis and healthy controls. Grouped bar chart showing estimated marginal means (±standard error) for three capillaroscopic parameters, derived from analysis of covariance (ANCOVA) with age as a covariate. Capillary length score was significantly lower in the atopic dermatitis group compared to healthy controls (F = 15.530, *p* < 0.001), indicating a higher prevalence of shortened capillaries (<100 µm). Tortuous capillaries (>10%) were significantly more prevalent in the AD group on unadjusted analysis (Fisher’s exact test *p* = 0.04); however, this difference showed only a trend toward significance after controlling for age (F = 3.188, *p* = 0.07). No difference was observed for crossing capillaries (F = 0.192, *p* = 0.662). Error bars represent standard errors of the estimated marginal means. Blue bars: atopic dermatitis group (*n* = 71); salmon bars: healthy control group (*n* = 71). ns: not significant; *** *p* < 0.001.

When age was included in the model as a covariate, no significant effect of age on capillary length was found (F = 0.501, *p* = 0.480). In contrast, the effect of the group variable on capillary length was found to be statistically significant (F = 15.53, *p* < 0.001). Differences in nailfold capillaroscopy findings between groups were evaluated using analysis of covariance (ANCOVA) after adjustment for age, which was included in the model as a covariate (months). The adjusted mean values (±standard error) for each capillaroscopic parameter are presented for the atopic dermatitis and healthy control groups. Capillary length was significantly lower in the AD group compared to healthy controls after controlling for age (F = 15.530, *p* < 0.001). No statistically significant differences were observed between the groups for tortuous capillaries (F = 3.188, *p* = 0.076) or crossing capillaries (F = 0.192, *p* = 0.662) (Table 2) (Figure 5). Age had no significant effect on the dependent variable (*p* = 0.8). Levene test showed that homogeneity of variance was achieved (*p* = 0.296).

**Figure 6:**
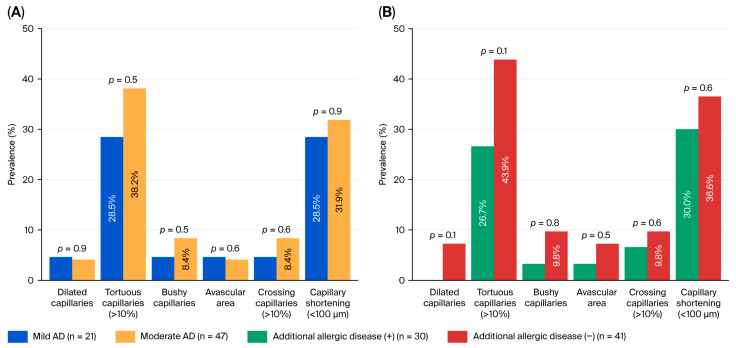
Distribution of nailfold capillaroscopy findings according to disease severity and allergic comorbidity in children with atopic dermatitis. (**A**) Comparison of NFC findings between mild (SCORAD ≤ 25, *n* = 21) and moderate (SCORAD 25–50, *n* = 47) AD. No statistically significant differences were observed for any capillaroscopic parameter (all *p* > 0.05), suggesting that microvascular remodeling occurs independently of clinical disease severity. (**B**) Comparison of NFC findings between AD patients with (*n* = 30) and without (*n* = 41) additional allergic diseases (allergic rhinitis, asthma, urticaria). No significant differences were found (all *p* > 0.05), indicating that capillaroscopic changes are not influenced by allergic comorbidity.

Similar capillary-scopic findings were observed in AD patients with and without additional allergic diseases (Table 3). According to SCORAD index, no significant change was observed in NFC findings in mild and moderate atopic dermatitis. A notable finding of our study was the lack of significant differences in capillary parameters between mild and moderate AD (*p* > 0.05). Capillary shortening, the most prominent finding in our cohort, was present in similar proportions in mild (28.5%) and moderate (31.9%) AD (*p* = 0.9), and tortuous capillaries (28.5% vs. 38.2%, *p* = 0.5) and bushy capillaries (4.7% vs. 8.4%, *p* = 0.5) were similarly distributed. The findings are summarized in Table 3 (Figure 6).

A substantial proportion of the study population with atopic dermatitis (47 of 71 patients, 66.1%) was categorized as having moderate disease severity based on the SCORAD index (25–50). Due to the very small number of severe AD cases (*n* = 3), no statistical analysis was performed for this subgroup. However, as an exploratory observation, capillary shortening (<100 µm) was present in all three severe AD patients (3/3, 100%), compared with 29.6% (8/27) in mild and 28.6% (10/35) in moderate AD.

When comparing patients with and without pathological findings in NFC, no statistically significant differences were observed in the presence of allergic sensitization, total IgE levels and eosinophil count.

To contextualize our findings within the broader spectrum of connective tissue diseases, published NFC patterns in studies of systemic sclerosis (SSc), dermatomyositis (DM), and systemic lupus erythematosus (SLE) were narratively compared with our original AD findings (Table 4). Given the heterogeneity of the referenced studies, this comparison should be interpreted as a qualitative narrative synthesis rather than a formal quantitative analysis. Dilated and giant capillaries, microhemorrhages, and tortuous capillaries are commonly reported findings in SSc, DM, and SLE. In contrast, the NFC profile identified in our AD cohort was fundamentally distinct from those reported in connective tissue diseases. The predominant finding in AD was capillary shortening (<100 µm), which was significantly more frequent than in healthy controls (33.8% vs. 8.4%, OR = 5.53, *p* < 0.001). Although capillary shortening has been described as part of the microangiopathic pattern in connective tissue diseases, it is not regarded as a characteristic or predominant feature of any specific connective tissue disease Notably, two hallmark features of scleroderma-spectrum microangiopathy giant capillaries and microhemorrhages were completely absent in our AD cohort. In contrast, both findings have been reported in all three connective tissue diseases (Table 4) [18,19,20,21,22].

### 3.2. Treatment

All patients used a topical moisturizing cream. Topical steroids were used in 48 (67.6%) patients, local mupirocin in 33 (46.5%) patients, topical tacrolimus in 9 (12.7%) patients, and immunotherapy in 1 (1.4%) patient. Omalizumab was not used in any of the patients.

## 4. Discussion

This comprehensive study aims to contribute to the understanding of the pathophysiology of atopic dermatitis by evaluating microvascular changes in NFC in children with atopic dermatitis. An inclusive capillaroscopy analysis of microvascular issues in children with atopic dermatitis could enhance our understanding of the disease’s pathophysiology, facilitate the identification of prognostic factors, and inform the development of effective treatment strategies. However, the limited number of studies on normal capillaroscopic reference ranges in healthy children and adolescents increases the importance of research in this area [23]. Our findings demonstrate that the AD group exhibited characteristic microvascular alterations compared with healthy controls. Reduced capillary length emerged as the most robust discriminating finding. Tortuous capillaries were observed more frequently in the atopic dermatitis group; however, this association did not retain statistical significance after age adjustment, suggesting a trend rather than a definitive finding. The presence of thickened and tortuous blood vessels in the skin of patients with AD has been reported in the literature. These changes may be associated with increased blood flow [12]. Similarly, a study by Arslan et al. reported increased tortuosity in capillaroscopic examination in pediatric patients with AD [8]. This may suggest that inflammation can lead to either adaptive or pathological changes in the microvasculature.

Additionally, the shortening of capillary length (<100 micrometers) was more pronounced in patients with AD compared to the HG. This finding suggests an impairment in the structural integrity of the microvascular bed in AD. Although there are few patients with severe AD, the significant reduction in capillary length observed in these patients suggests that this change may become more pronounced as the disease progresses.

In the study by Arslan et al., a decrease in capillary density and an increase in capillary dilatation were detected [8]. In our study, no significant difference was found between AD and healthy controls in terms of dilated capillaries. Cutolo et al. described capillary dilatation as a component of the scleroderma pattern, representing a local autoregulatory response to tissue hypoxia and an early sign of vessel wall damage [24]. The fact that dilated capillaries in our AD cohort were not only infrequent but also less prevalent than in healthy controls suggests that the hypoxia-driven endothelial injury mechanism characteristic of scleroderma-spectrum disorders does not play a prominent role in the microvascular pathology of AD. Furthermore, no correlation was found between prolonged follow-up duration of atopic dermatitis and capillary change.

Bushy or branched capillaries are considered indicative of vascular damage or neoangiogenesis, particularly in other connective tissue diseases such as systemic sclerosis [25]. Therefore, the presence of such capillary morphology in AD may be an indicator of vascular remodeling from the subclinical inflammatory stage to the advanced stages of the disease. In our series, bushy capillaries were remarkable in the AD group compared to the HG. These findings suggest that microvascular remodeling in AD follows a fundamentally different pathophysiological pathway compared to scleroderma spectrum disorders. While endothelial destruction is prominent in connective tissue diseases, the changes observed in AD likely reflect a non-destructive vascular remodeling process triggered by chronic Th2 inflammation and Vascular Endothelial Growth Factor (VEGF)-mediated angiogenesis [10]. The combination of capillary shortening and bushy capillaries in the absence of giant capillaries, microhemorrhage, and significant capillary loss may represent a microvascular phenotype specific to AD and warrant further investigation as a potential biomarker of microvascular involvement.

In our study, no microhemorrhages were observed in the atopic dermatitis group, whereas microhemorrhages were detected in the healthy control group. First, low-frequency microhemorrhages may also be detected in healthy children. A study conducted in healthy individuals reported that 5.33% may exhibit microhemorrhages [26]. Similarly, Kavrul Kayaalp et al. documented microhemorrhages in 1.5% of healthy pediatric controls [27]. The prevalence of microhemorrhages in our healthy control group (8.4%) was higher than that reported by Dundar et al. in a large multicenter cohort of healthy children (2.7%) [17]. However, this difference may be attributable to sampling variability related to the relatively small cohort size. Given the low event counts and the absence of microhemorrhages in the AD group, these findings should be interpreted cautiously. Notably, Jakhar et al. reported a microhemorrhage prevalence of 14% in healthy adults [28], demonstrating that overall prevalence ranges are broader than pediatric data alone suggest. Bushy capillaries (observed exclusively in AD) and microhemorrhages (observed exclusively in healthy controls) reached statistical significance on Fisher’s exact test. However, the wide confidence intervals of the Haldane–Anscombe corrected odds ratios reflect the inherent instability of effect size estimation under zero-cell conditions. Therefore, although Fisher’s exact test identified these differences as significant, the precision of the effect size estimates remains limited; both findings should be considered exploratory.

Several pathophysiological mechanisms may explain the absence of hemorrhages in the AD group. Chronic inflammation in AD leads to vascular remodeling characterized by thickened capillary walls and structurally altered vessels, which may paradoxically increase resistance to capillary rupture [12]. The marked capillary shortening observed in our AD patients may also reflect a distinct microvascular bed with different hemodynamic properties. Leung et al. demonstrated that IL-4/IL-13 signaling disrupts vascular endothelial barrier function, leading to leakage of plasma proteins into AD skin [29]. This finding suggests that vascular dysfunction in AD predominantly manifests as increased vascular permeability to plasma proteins clinically reflected by exudation and edema rather than structural capillary rupture resulting in erythrocyte extravasation and microhemorrhage. In AD, a proangiogenic milieu may promote neovascularization, as reflected by the presence of bushy capillaries. The absence of destructive capillaroscopic findings suggests that these changes may represent a non-destructive microvascular remodeling process rather than the vascular injury typically observed in connective tissue diseases [25]. Nevertheless, larger studies are needed to clarify further the relationship between microhemorrhages and disease-specific microvascular alterations in AD.

Furthermore, no differences were found between healthy controls and AD patients in terms of mean capillary counts, dilated capillaries, giant capillaries, avascular areas, or crossed capillaries. However, a comprehensive study evaluating NFC findings in healthy children reported that in each age group, there were varying percentages of dilate capillaries (8.7%), capillary tortuosity (14.4%), crossed capillaries (43.1%), microhemorrhage (2.7%), and avascular area (4.8%). It was emphasized, however, that giant or bushy capillaries were not observed in the HG [17]. These findings may suggest that some capillary parameters do not represent characteristic microvessel changes in AD, or that changes in these parameters are not specific to AD. However, different studies have reported a decrease in capillary density and an increase in avascular areas in patients with AD [8]. The most significant reason for this difference in our study may be the larger number of patients with severe atopic dermatitis in the other study.

In our study, no significant correlation was found between NFC findings and the SCORAD index in patients with mild and moderate AD. However, in our study, although not statistically significant, an increase in the frequency of tortuous capillaries, bushy capillaries, crossing capillaries, and shortening of capillary length was found in the moderate atopic dermatitis group. However, it cannot be said that capillaroscopic findings are always directly related to SCORAD index. This observation has important pathophysiological implications, suggesting that microvascular remodeling in AD is not a severity-dependent phenomenon but rather an early event established during the initial stages of the disease that does not intensify proportionally with clinical worsening. This pattern differs from systemic sclerosis, in which capillaroscopic abnormalities follow a progressive early–active–late pattern associated with disease duration and organ involvement [18,30]. The absence of a severity-related gradient in AD supports the hypothesis that the underlying mechanism is fundamentally distinct from the progressive endothelial destruction observed in scleroderma-spectrum disorders. Instead, it may reflect a Th2-mediated vascular remodeling process that begins early in the disease course and persists throughout disease progression. These findings suggest that the microvascular changes observed in AD may be driven by a distinct pathological process rather than by the presence of a broader atopic predisposition or systemic allergic inflammation.

The very small number of patients in the severe AD subgroup (*n* = 3) precludes any statistical conclusions regarding this subgroup. This underrepresentation was largely attributable to the study’s exclusion criteria, which required the absence of active dermatitis on the hands and nailfold region and excluded patients receiving systemic immunosuppressive therapy, conditions that frequently apply to severe AD patients. Nevertheless, the observation that capillary shortening was present in all three severe AD patients (100%), compared with approximately 29% in both mild and moderate AD, is a noteworthy exploratory finding.

Our study consisted of 36 patients (51.4%) with pathological NFC findings and 35 patients (48.6%) with normal NFC findings. No statistically significant differences were observed between patients with pathological and normal NFC findings in terms of SCORAD index, eosinophil count, total IgE levels, immunoglobulin levels, or allergic sensitization. These findings suggest that the presence of pathological NFC findings does not appear to correlate with the clinical severity of atopic dermatitis, humoral immunity, the presence of an allergic phenotype, total IgE levels, or elevated eosinophil counts. It is thought that pathological NFC findings may reflect pathophysiological mechanisms belonging to a distinct phenotype of atopic dermatitis, developing independently of eczema severity and increases in serum biomarker levels (eosinophil count and total IgE). Supporting this hypothesis, some studies have shown that specific skin pathologies do not always directly correlate with overall AD severity scores [12,31]. For instance, a study by Sabău et al. reported that specific ultrasonographic parameters determined by high-frequency ultrasonography did not correlate with SCORAD index [32]. This situation strengthens the idea that microvascular changes in the skin may represent distinct pathological processes that are not reflected in the overall clinical appearance of the disease or in systemic biomarkers [32]. In the study by Bakker et al., it is emphasized that a single biomarker is insufficient to comprehensively reflect the disease process, given that atopic dermatitis is a heterogeneous disease with variability over time [33]. Therefore, nailfold capillaroscopic evaluation may represent a potential non-invasive adjunctive approach that could be considered for inclusion in future biomarker assessment strategies. The limitations of this study include its single-center design, the performance of nailfold capillaroscopic evaluations by a single observer, and the absence of inter- or intra-observer reliability analyses.

## 5. Conclusions

This study demonstrates characteristic nailfold capillaroscopic changes in children with atopic dermatitis, with reduced capillary length emerging as the most robust discriminating finding. A trend toward increased tortuous capillaries was also observed; however, this did not retain statistical significance after age adjustment. Giant capillaries, hallmark features of connective tissue disease–associated microangiopathy, were not observed in either group. Bushy capillaries (observed exclusively in the AD group) and microhemorrhages (observed exclusively in healthy controls) reached statistical significance on Fisher’s exact test but were associated with wide confidence intervals due to zero-cell counts; therefore, both findings should be considered exploratory. Our findings suggest a potential role of microvascular remodeling and neoangiogenesis in the pathophysiology of atopic dermatitis. Notably, NFC findings showed no significant correlation with disease severity and were independent of allergic comorbidities, suggesting that the observed vascular remodeling may represent a component of the core pathophysiological mechanisms of AD rather than a secondary process related to disease severity or general atopic predisposition. To our knowledge, this is one of the first studies to comprehensively characterize the capillary phenotype of childhood AD and demonstrate a non-destructive microvascular remodeling pattern distinct from both connective tissue diseases and healthy controls. Elucidation of the molecular mechanisms underlying these microvascular changes observed in atopic dermatitis and evaluation of their effects on disease prognosis are important for future research aimed at developing novel therapeutic strategies and suggest that nailfold capillaroscopy may be used as a non-invasive method for assessing microvascular alterations in children with AD.

## Figures and Tables

**Table 1:** Nailfold capillaroscopy findings.

	Atopic Dermatitis N = 71 (33 F/38 M)	Healthy Group N = 71 (40 F/31 M)	*p*
Mean capillary count ± SD (years)	9.6 ± 1.7	10.6 ± 1.3	0.07
Dilated capillary (*n*, %)	3 (4.2%)	7 (9.8%)	0.3
Giant capillary (*n*, %)	0	0	
Tortuous capillary > 10% (*n*, %)	26 (36.6%)	16 (22.5%)	0.04
Microhemorrhage (*n*, %)	0	6 (8.4%)	0.02
Bushy capillary (*n*, %)	6 (8.4%)	0	0.01
Avascular area (*n*, %)	4 (5.6%)	2 (2.8%)	0.4
Crossing capillary > 10% (*n*, %)	6 (8.4%)	8 (11.2%)	0.7
Shortening of capillary length (<100 μm) (*n*, %)	24 (33.8%)	6 (8.4%)	<0.01

SD: standard deviation.

**Table 2:** Age-related intergroup capillaroscopy findings (ANCOVA Results).

	Atopic Dermatitis (Mean ± SE)	Healthy (Mean ± SE)	F (Group)	*p*	R^2^
>10% Tortuous capillaries	0.364 ± 0.054	0.227 ± 0.054	3.188	0.076	0.027
Capillary length (<100 μm)	0.70 ± 0.060	1.03 ± 0.059	15.53	<0.001	0.102
Crossing increase	0.088 ± 0.036	0.110 ± 0.036	0.192	0.662	0.011

SE: standard error; F: F statistic representing the group effect in the age-adjusted ANCOVA model; *p*: *p* value for intergroup differences; R^2^: proportion of variance explained by the group effect.

**Table 3:** NFC findings according to SCORAD index and additional allergy disease.

	Mild Atopic Dermatitis *n* = 21	Moderate Atopic Dermatitis *n* = 47	*p*	Additional Allergy Disease Yes (*n* = 30)	Additional Allergy Disease No (*n* = 41)	*p*
Mean capillary count ± SD (years)	9.4 ± 1.5	10 ± 1.8	0.07	9.5 ± 1.8	9.7 ± 1.6	0.6
Dilated capillary (*n*, %)	1 (4.7%)	2 (4.2%)	0.9	0	3	0.1
Giant capillary (*n*, %)	0	0				
Tortuous capillary > 10% (*n*, %)	6 (28.5%)	18 (38.2%)	0.5	8	18	0.1
Microhemorrhage (*n*, %)	0	0				
Bushy capillary (*n*, %)	1 (4.7%)	4 (8.4%)	0.5	1	4	0.8
Avascular area (*n*, %)	1 (4.7%)	2 (4.2%)	0.6	1	3	0.5
Crossing capillary > 10% (*n*, %)	1 (4.7%)	4 (8.4%)	0.6	2	4	0.6
Shortening of capillary length (<100 μm) (*n*, %)	6 (28.5%)	15 (31.9%)	0.9	9	15	0.6

NFC: Nailfold capillaroscopy, SD: standard deviation, SCORAD: SCORing of atopic dermatitis.

**Table 4:** Comparative overview of nailfold capillaroscopy findings in atopic dermatitis (present study) and connective tissue diseases: a narrative synthesis based on published literature.

NFC Finding	AD (Present Study)	SSc	DM	SLE
Capillary density ↓	+	+++	++	+
Dilated capillaries	+	+++	++	+
Giant capillaries	−	+++	++	+
Tortuous capillaries	++	+	+	++
Bushy/capillaries	+	++	+	+
Microhemorrhage	−	+++	++	++
Avascular areas	+	+++	+	+
Crossing capillaries	+	+	+	+
Capillary shortening (<100 µm)	+++	+	+	+

− absent (0%); + present/mild; ++ frequent; +++ characteristic/dominant finding of the disease. AD: atopic dermatitis; SSc: systemic sclerosis; DM: dermatomyositis; SLE: systemic lupus erythematosus; NFC: nailfold capillaroscopy. The AD column is based on original data from the present study. SSc, DM, and SLE grading represent narrative synthesis derived from published literature and should not be interpreted as a direct quantitative comparison.

## Data Availability

The data that support the study findings are available from the corresponding author upon reasonable request.

## References

[1] Ab Hadi H., Tarmizi A.I., Khalid K.A., Gajdács M., Aslam A., Jamshed S. (2021). The Epidemiology and Global Burden of Atopic Dermatitis: A Narrative Review. Life.

[2] Gavrilita E., Silion S.I., Bitca M.L., Tatu A.L. (2024). Insights into Intrinsic Atopic Dermatitis: Immunogenicity, Dysbiosis, and Imaging (Reflectance Confocal Microscopy, Optical Coherence Tomography). Clin. Cosmet. Investig. Dermatol..

[3] Tian J., Zhang D., Yang Y., Huang Y., Wang L., Yao X., Lu Q. (2024). Global epidemiology of atopic dermatitis: A comprehensive systematic analysis and modelling study. Br. J. Dermatol..

[4] Langan S.M., Mulick A.R., Rutter C.E., Silverwood R.J., Asher I., García-Marcos L., Ellwood E., Bissell K., Chiang C., El Sony A. (2023). Trends in eczema prevalence in children and adolescents: A Global Asthma Network Phase I Study. Clin. Exp. Allergy.

[5] Egawa G., Kabashima K. (2018). Barrier dysfunction in the skin allergy. Allergol. Int..

[6] Radi G., Campanti A., Diotallevi F., Martina E., Marani A., Offidani A. (2022). A systematic review of atopic dermatitis: From physiopathology to treatment. Biomedicines.

[7] Grafanaki K., Bania A., Kaliatsi E.G., Vryzaki E., Vasilopoulos Y., Georgiou S. (2023). The Imprint of Exposome on the Development of Atopic Dermatitis across the Lifespan: A Narrative Review. J. Clin. Med..

[8] Arslan Uku S., Demir B., Cicek D., Inan Yuksel E. (2021). Assessment of nail findings in children with atopic dermatitis. Clin. Exp. Dermatol..

[9] Du F., Wu H., Xue J., Yao S., Li R., Zhang J., Wang H., Cheng Q., Zhao F., Huang Y. (2025). Long-term investigation of 3D tissue detailed structure and multiparametric vascular network properties using OCT/OCTA for guiding atopic dermatitis theranostics. J. Transl. Med..

[10] Lee H.J., Hong Y.J., Kim M. (2021). Angiogenesis in Chronic Inflammatory Skin Disorders. Int. J. Mol. Sci..

[11] Varricchi G., Granata F., Loffredo S., Genovese A., Marone G. (2015). Angiogenesis and lymphangiogenesis in inflammatory skin disorders. J. Am. Acad. Dermatol..

[12] Tsutsumi M., Fukuda M.B., Kumamoto J.B., Goto M., Denda S., Yamasaki K., Aiba S., Nagayama M., Denda M. (2016). Abnormal Morphology of Blood Vessels in Erythematous Skin from Atopic Dermatitis Patients. Am. J. Dermatopathol..

[13] Leung D.Y., Boguniewicz M., Howell M.D., Nomura I., Hamid Q.A. (2004). New insights into atopic dermatitis. J. Clin. Investig..

[14] Deegan A.J., Wang R.K. (2019). Microvascular imaging of the skin. Phys. Med. Biol..

[15] Hanifin J.M., Rajka G. (1980). Diagnostic Features of Atopic Dermatitis. Acta Derm.-Venereol..

[16] Melsens K., Cutolo M., Schonenberg-Meinema D., Foeldvari I., Leone M.C., Mostmans Y., Badot V., Cimaz R., Dehoorne J., Deschepper E. (2022). Standardized nailfold capillaroscopy in children with rheumatic diseases: A worldwide study. Rheumatology.

[17] Dundar H.A., Adrovic A., Demir S., Demir F., Cakmak F., Ayaz N.A., Sözeri B., Bilginer Y., Kasapçopur O., Unsal E. (2024). Description of the characteristics of the nailfold capillary structure in healthy children: A multicentric study. Rheumatology.

[18] Cutolo M., Sulli A., Pizzorni C., Accardo S. (2000). Nailfold videocapillaroscopy assessment of microvascular damage in systemic sclerosis. J. Rheumatol..

[19] Dima A., Berza I., Popescu D.N., Parvu M.I. (2021). Nailfold capillaroscopy in systemic diseases: Short overview for internal medicine. Rom. J. Intern. Med..

[20] Schonenberg-Meinema D., Bergkamp S.C., Nassar-Sheikh Rashid A., van der Aa L.B., de Bree G.J., Cate R.T., Cutolo M., Hak A.E., Muller P.C.H., van Onna M. (2021). Nailfold capillary abnormalities in childhood-onset systemic lupus erythematosus: A cross-sectional study compared with healthy controls. Lupus.

[21] Bărbulescu A.L., Vreju A.F., Bugă A.M., Sandu R.E., Criveanu C., Tudoraşcu D.R., Gheonea I.A., Ciurea P.L. (2015). Vascular endothelial growth factor in systemic lupus erythematosus—Correlations with disease activity and nailfold capillaroscopy changes. Rom. J. Morphol. Embryol..

[22] Sundaray S., Mishra S., Dash S.C., Sundaray N.K. (2022). Nailfold Videocapillaroscopy in Connective Tissue Diseases with Raynaud’s Phenomenon in an Indian Population. Rambam Maimonides Med. J..

[23] Terreri M.T., Andrade L.E., Puccinelli M.L., Hilário M.O., Goldenberg J. (1999). Nailfold capillaroscopy: Normal findings in children and adolescents. Semin. Arthritis Rheum..

[24] Cutolo M., Pizzorni C., Secchi M.E., Sulli A. (2008). Capillaroscopy. Best Pract. Res. Clin. Rheumatol..

[25] Dogdu M., Altinyazar H.C., Yilmaz S., Demirbas A., Diremsizoglu E. (2024). Dermatoscopic assessment of nailfold capillary structures in connective tissue diseases. Arch. Dermatol. Res..

[26] Gorasiya A., Mehta H., Prakashey A., Dave M. (2022). Nailfold capillaroscopy of healthy individuals—An observational study. Indian Dermatol. Online J..

[27] Kavrul Kayaalp G., Arık S.D., Akgün Ö., Menentoğlu B., Doğru A., Çakmak F., Aktay Ayaz N. (2026). Cold fingers under the lens: Unveiling microvascular differences between children with primary Raynaud’s phenomenon and healthy individuals. Mod. Rheumatol..

[28] Jakhar D., Grover C., Kaur I. (2020). Nailfold capillaroscopy with USB dermatoscope: A cross-sectional study in healthy adults. Indian J. Dermatol. Venereol. Leprol..

[29] Leung D.Y., Bissonnette R., Kreimer S., Berdyshev E., Bafna S., Lyubchenko T., Richers B.N., Garcia S., Ramirez-Gama M., Hall C.F. (2023). Dupilumab Inhibits Vascular Leakage of Blood Proteins into Atopic Dermatitis Skin. J. Allergy Clin. Immunol. Pract..

[30] Sulli A., Paolino S., Pizzorni C., Ferrari G., Pacini G., Pesce G., Carmisciano L., Smith V., Cutolo M. (2019). Progression of nailfold capillaroscopic patterns and correlation with organ involvement in systemic sclerosis: A 12 year study. Rheumatology.

[31] Byers R.A., Maiti R., Danby S.G., Pang E.J., Mitchell B., Carré M.J., Lewis R., Cork M.J., Matcher S.J. (2017). Characterizing the microcirculation of atopic dermatitis using angiographic optical coherence tomography. Proc. SPIE Int. Soc. Opt. Eng..

[32] Sabău M., Boca A.N., Ilies R.F., Tătaru A. (2018). Potential of high-frequency ultrasonography in the management of atopic dermatitis. Exp. Ther. Med..

[33] Bakker D.S., de Bruin-Weller M., Drylewicz J., van Wijk F., Thijs J.L. (2023). Biomarkers in atopic dermatitis. J. Allergy Clin. Immunol..

